# Unlocking optimism in everyday life: a short-term study on the power of live comedy to reduce stress and anxiety in general public

**DOI:** 10.1080/21642850.2025.2493141

**Published:** 2025-04-24

**Authors:** Toshiko Tomisawa, Kayo Horie, Naoya In, Naoki Nanashima, Shizuka Takamagi, Kasumi Mikami

**Affiliations:** a Department of Nursing Sciences, Hirosaki University Graduate School of Health Sciences, Aomori Prefecture, Japan; b Department of Medical Technology, Hirosaki University Graduate School of Health Sciences, Aomori Prefecture, Japan; c Department of Nutrition, Faculty of Health Sciences, Aomori University of Health and Welfare, Aomori Prefecture, Japan

**Keywords:** Laugh, optimism, pessimism, salivary alpha-amylase, comedy live performance

## Abstract

**Background:**

Although there have been many studies on laughter and health, few studies have clarified the effects of laughter on optimism and pessimism, as well as anxiety. The purpose of the study was to evaluate the effects of viewing a comedy live performance on optimism, pessimism, and anxiety, sAA as a stress marker, and examine the relationship between laughter attitude and optimism, pessimism and stress marker and clarify significant factors that contribute to optimism.

**Methods:**

In this pre – post study, we evaluated the effects on attendees of laughter elicited by a comedy live performance (CLP). The participants were 110 volunteers aged 18–64 years. Participants attended a two-hour CLP by four famous Japanese comedians (Sisonnu, Tonikaku Akarui Yasumura, Mouchugakusei, and Panther) and completed pre – and post-event questionnaires that included items from the Japanese Optimism and Pessimism Scale (JOPS), State-Trait Anxiety Inventory (STAI), the Laughter Attitude Scale (LAS) and Salivary alpha-amylase (sAA).

**Results:**

The valid response rate was 101. Participants’ optimism was higher, and pessimism, anxiety and sAA were lower, after attending the CLP. α-amylase was significantly lower in more people before and after CLP. Factors contributing to optimism were strongly influenced by the degree to which people made them laugh, as well as pessimism, anxiety, and forced laughter.

**Conclusion:**

The personal factors contributing most strongly to optimism were ‘A state that makes you laugh,’ as well as pessimism, anxiety, and a low level of ‘Forced Laughter.’ Attending a two-hour CLP increased optimism and decreased stress level, pessimism and anxiety, these effects were more pronounced in those who laughed regularly in their everyday lives.

## 1 Introduction

In today’s society, quality of life has been affected by stress caused by competition with others, various types of relationships, and significant changes on a global scale. One way to improve quality of life is through laughter. Laughter can reduce social tension, demonstrate to others a feeling of trust and absence of threat (Wood & Niedenthal, 2018), and smooth friction between people. Laughter encourages positive feelings and is an effective and healthy way to overcome stress (Yim, 2016). Numerous researchers and practitioners have demonstrated various positive effects of laughter on human health, which include improving breathing, stimulating circulation, decreasing stress hormones, increasing immune system defenses, increasing the pain threshold and tolerance, and enhancing mental function (Martin, 2004; Mora-Ripoll, 2008; Sahakian, 2007; Yoshino SF & Kohda, 1996). Laughter is also an integral part of human life and communication, and has been shown to prevent negative emotions and enhance well-being (Gonot-Schoupinsky & Garip, 2018). Laughter intervention programs have been found to promote increased optimism, positive thinking, and subjective well-being (Scheier & Carver, 1992; Stanton et al., 2002). In addition, recent years, methods have been used to objectively evaluate stress by measuring cortisol and amylase activity in saliva rather than in blood. In particular, salivary α-amylase activity (sAA) has a faster response to stress than salivary cortisol and is attracting attention as an index for detecting changes in sympathetic nerve activity (Takai N et al., 2004).

Characteristic optimism, or dispositional optimism, is defined as the tendency to expect positive outcomes (Scheier et al., 1989) and has been shown to be associated not only with physical health as well as adjustment and mental health. For example, people with high optimism have better health than those with low optimism (Michael F. Scheier CSC. Optimism, Coping, 1985) and have less depression after stressful events (Carver & Gaines, 1987). It has been shown that those with high optimism recover physically and return to normal life after discharge following coronary artery bypass surgery more quickly compared to those with low optimism (Carver & Gaines, 1987), and have higher subjective wellbeing and quality of life (QOL) at six months and five years after discharge. The effect of laughter on optimism may differ depending on one’s attitude toward laughter, according to one’s usual preference and style among the various types of laughter; however, there have been few studies regarding the relationship between attitude toward laughter and optimism. We believe that clarifying the relationship between laughter attitude and optimism could inform interventions tailored to the individual in terms of the relationship between the type of laughter and mental health. However, there are also few studies for laughter that have evaluated stress using sAA as a sympathetic nervous system index (Meier et al., 2021; Sakurai et al., 2019). Furthermore, few studies have examined the effects of laughter from multiple perspectives, including not only physiological changes but also psychological changes.

## 2 Methods

### 2.1 Aim

The aim of this study is to evaluate the effects of viewing a comedy live performance on optimism, pessimism, and anxiety, sAA as a stress marker, and examine the relationship between laughter attitude and optimism, pessimism and stress marker and clarify significant factors that contribute to optimism.

### 2.2 Participants

The participants were recruited from people aged 18–64 years who attended a CLP in Hirosaki, Aomori, Japan, in July 2023. The exclusion criterion was inability to understand Japanese.

### 2.3. Ethics statement

Participants were advised orally and in written form of the purpose and outline of the study, that the survey information would not be used for any purpose other than this study, that participation was voluntary with no disadvantage incurred depending on whether or not to cooperate. All participants provided written informed consent prior to participation in the study. Personal information was anonymized by replacing the name information with a number. The study was approved by the Institutional Review Board of Hirosaki University School of Health Sciences (approval No. 2023-012).

## 3 Procedure

### 3.1 Study design and setting

A pre – post design was used to evaluate the effects of laughter induced by a CLP. It was concluded at Hirosaki city hall on September 9, 2023. Participants were informed of the study purpose, methods, risks, and benefits, and provided written informed consent. Participants were asked to arrive early at the venue to complete a pre-CLP questionnaire. They also completed a post-CLP questionnaire. Each participant received an honorarium after all pre – and post-CLP research data had been collected.

### 3.2 Comedy live performance

Four famous Japanese comedians performed their routines at a two-hour CLP that was organized by the Division of Cultural Promotion in Hirosaki City with the aim of supporting citizens’ health through comedy entertainment as a form of culture. The comedians were Sisonnu, Tonikaku Akarui Yasumura, Mouchugakusei, and Panther.

### 3.3 Salivary sample collection

The participants were asked not to drink or eat 1 hour before the experiment. Saliva collections were used the passive drool method with the Saliva Collection Aid (SCA), an assisting tool for collecting the whole saliva. There were using the Saliva Collection Aid (Funakoshi, Tokyo, Japan) and immediately stored on ice. And then stored at – 20°C until analysis.

### 3.4 Data sources/measurement

Data were collected as variables, including Socio-demographic characteristics (age, gender, occupations, preference of comedy, how many times laughter in a day, et al.), how fun of CLP, satisfaction of CLP, scores of Laughter Attitude Scale, scores of Japanese Optimism and Pessimism Scale, STAI and sAA.

### 3.5 Salivary alpha-amylase (sAA) activity

sAA activity was measured using a salivary α-amylase kinetic enzyme assay kit (Funakoshi, Tokyo, Japan) according to manufacturer’s protocol. In briefly, on day of assay, thaw the saliva samples completely, vortex, centrifuged at 1500 × g for 15 min. Next an overall 1:200 dilution was achieved in two serial steps. For assay, 1:200 diluted each saliva sample 8 μl was to individual wells in a 37°C preheated. And then a multichannel pipette was used to simultaneously add 320 μl of the 37°C preheated substrate into each well. Then, reading was performed on the Multimode Microplate Readers TriStar LB 941 (Berthold Japan, Tokyo, Japan) the Optical Density (OD) in 405 nm at a temperature of 37°C. Read OD at exactly1 and 3 min and Calculation of enzyme activity was subtracting the one-minute reading from the three-minute reading and multiplied by the conversion factor.

### 3.6 Laughter attitude scale (LAS)

The Laughter Attitude Scale is a 19-item scale that measures how a person habitually tends to laugh and uses a 5-point scale ranging from 5: totally agree to 1: totally disagree (Fukushima & Hashimoto, 2014). The subscale contains 7 items for ‘A state that makes you laugh,’ 8 items for ‘Actively making others laugh,’ and 4 items for ‘Forced laughter.’ This scale was included only in the pre-CLP questionnaire.

### 3.7 Japanese optimism and pessimism scale (JOPS)

The 20-item Japanese Optimism and Pessimism Scale (JOPS) (Toyama, 2012) was developed by Toyama to measure optimism and pessimism independently in the Japanese population. The scale uses a 4-point scale ranging from 4 (agree with the statement ‘I am optimistic’) to 1 (disagree with the statement ‘I am not pessimistic’). Pre – and post-measures were taken.

### 3.8 Japanese state-trait anxiety inventory (STAI)

The STAI is a scale developed by Shimizu et al. that measures anxiety state using a 4-point scale ranging from 4: completely true to 1: completely false. Pre and post measures were taken (Shimizu H, 1981).

### 3.9 Additional questionnaire items included questions regarding gender, age, and occupation

Before the event, participants were asked how often they usually laugh; and after the event, they were asked about their satisfaction with the CLP, how much they laughed, and whether they enjoyed the CLP.

### 3.10 Study size

Using eight selected independent variables to run multiple regression, this study needed a minimum sample size of 100 subjects to achieve 85% power and a medium effect size (.15) at α = .05.

### 3.11 Statistical analysis

All data were analyzed by the Statistical Package for the Social Sciences (SPSS) for Windows, version 26.0 (SPSS Inc., Armonk, NY, USA). A series of the Spearman r correlations, Wilcoxon rank sum test and Mann – Whitney U test was used to examine the associations between participant demographics and each of the four scales. Wilcoxon rank sum test was used to analyze the comparison of each scale before and after the CLP, and Spearman’s correlation coefficient was calculated for the correlation between each scale. Multiple linear regression was employed to determine which variables from the following contributed to variation in the level of optimism according to the Japanese Optimism and Pessimism Scale: demographics, pessimism according to the Japanese Optimism and Pessimism Scale, STAI, and Laughter Attitude Scale. The forced entry method was used to choose the final regression model. The α level was set at .05 for statistical significance.

## 4. Results

### 4.1 Socio-demographic characteristics

The valid response rate was 99% (n = 101). The backgrounds of the participants are summarized in Table 1. By gender, 78 (77.2%) were women. In terms of age, 1(1%) participants were in their teens, 7 (6.9%) of participants were in their twenties, 26 (25.7%) of participants were in their thirties, 32 (31.7%) in their forties, and 28 (27.7%) in their fifties; 65 (64.4%) were company employees, 15 (14.9%) were in other employment, and 9 (8.9%) were housewives. Regarding the frequency of laughing in daily life, 66 participants (65.3%) laughed several times a day and 25 (24.8%) laughed once a day (Table 2). Regarding participants’ impressions of the CLP, 96 (95%) enjoyed the performance very much and 91 (90.1%) said they had laughed a lot (Table 3).

**Table 1.** Background of participants (n = 101).

Personality	Items	Number	%
gender	Male	23	22.8
	Female	78	77.2
age	10s	1	1.0
	20s	7	6.9
	30s	26	25.7
	40s	32	31.7
	50s	28	27.7
	60s	7	6.9
Occupation	Student	7	6.9
	Employee	65	64.4
	Independent business	2	2.0
	Farming and Fishery	3	3.0
	Housemaker	9	8.9
	Other	15	14.9

**Table 2.** Usual frequency of laughing (n = 101).

	Number	%
Laugh many times a day.	66	65.3
Laugh once a day	25	24.8
Laugh several times a week	7	6.9
Laugh several times a month	3	3.0
I don’t laugh at all	0	0.0

**Table 3.** Results of a post survey after a comedy show (n = 101).

Question	Choice	number	%
How much fun was it?	I enjoyed it very much.	96	95
	I had a good time.	5	5
How much did you laugh	I laughed a lot.	91	90.1
	I laughed a couple of times	4	4.0
	I laughed moderately but not a lot	3	3.0
	I laughed some of the time	3	3.0
	unanswered	1	1.0

### 4.2 Reliability of the scales

The Laughter Attitude Scale showed high reliability, with the subscale ‘A state that makes you laugh’ at 0.79, ‘Actively make someone laugh’ at 0.82, and ‘Forced laughter’ at 0.74. Reliability was 0.94 for both the optimism and pessimism sub-scales of the Japanese Optimism and Pessimism Scale, and was 0.91 for STAI. Although only four items were scored for ‘Forced smile,’ which is somewhat low, each scale was scored for comparison.

### 4.3 Change in optimism score, pessimism score, STAI and sAA

**Figure 1.**
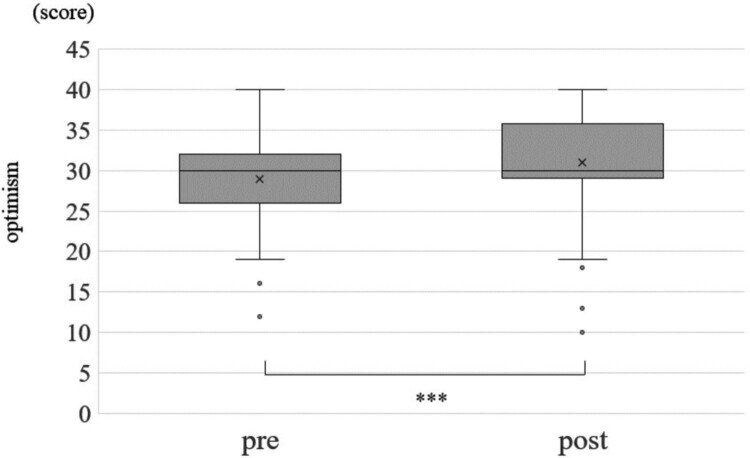
Change in optimism score of JOPS. Statistical Analysis: Wilcoxon rank sum test. ***p < 0.001.

**Figure 2.**
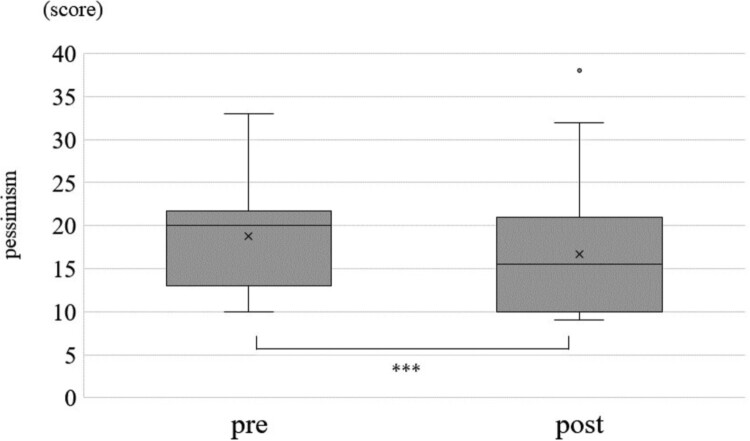
Change in pessimism score of JOPS. Statistical Analysis: Wilcoxon rank sum test. ***p < 0.001.

**Figure 3.**
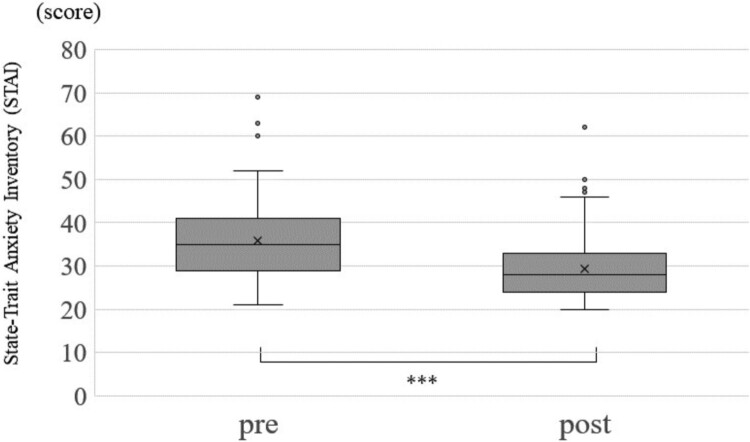
Change in State – Trait Anxiety Inventory (STAI) score. Statistical Analysis: Wilcoxon rank sum test. ***p < 0.001.

**Figure 4.**
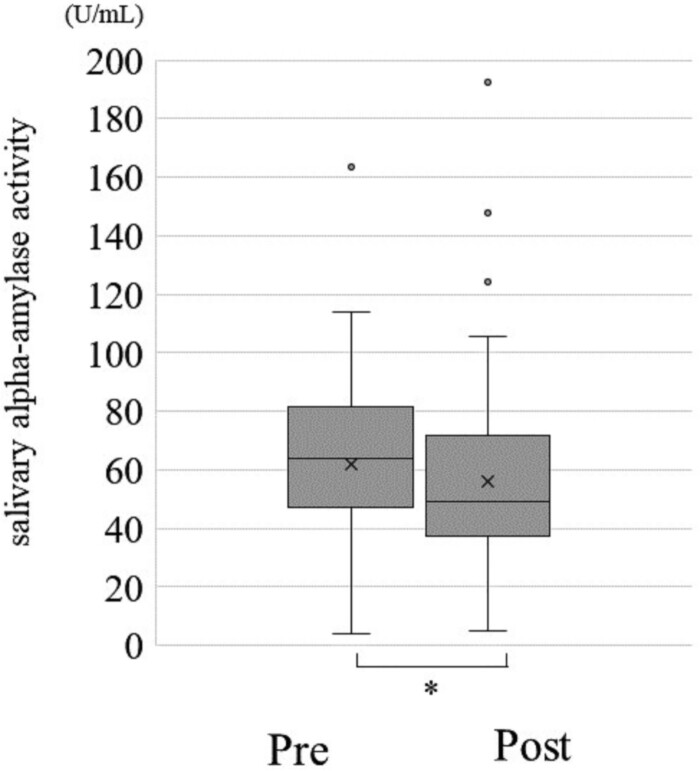
Over all changes in salivary alpha-amylase activity. Statistical Analysis: Mann – Whitney U test, *p < 0.05.

**Figure 5.**
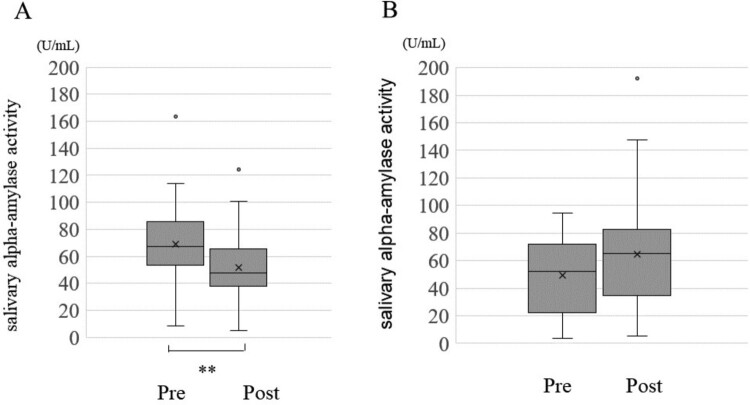
Comparison of changes in salivary alpha-amylase activity post CLP by increase or decrease group. A: Pre > Post group (n = 65), B: Pre < Post group (n = 37). Statistical Analysis: Mann – Whitney U test., **p < 0.001.

Change in each of the optimism score, pessimism score, STAI and sAA between before and after the CLP is shown in Figures 1–3, respectively. The average optimism score was 29.6 (Standard Division:SD = 6.1) before the CLP and increased significantly to an average of 30 (SD = 5.9) after the CLP (*p* < 0.001) (Figure 1, Table 4). Median pessimism score decreased significantly from 18 (SD = 5.9) before CLP to 17.6 (SD = 5.9) after CLP (*p* < 0.001) (Figure 2, Table 4). STAI score decreased significantly from 33.2 (SD = 9.3) before CLP to 32.5 (SD = 9.0) after CLP (*p* < 0.001) (Figure 3, Table 4). sAA was significantly decreased post-CLP than pre-CLP (*P* = 0.03) is shown in Figure 4. Furthermore, compared to pre sAA, post sAA showed a higher proportion of people who decreased (63.7%) than increased (36.3%) (data not shown). Figure 5A and Table 4 show that significant differences in pre > post group (*P* < 0.001), although, no significant difference was observed in the pre < post group (*P* = 0.07) (Figure 5B).

**Table 4.** Change in outcome index between pre and post CLP (n = 101).

	Pre (Mean ± SD)	Post(Mean ± SD)	*p*
Optimism (score)	29.6 ± 6.1	30.0 ± 5.9	*p* < 0.001
Pessimism (score)	18.0 ± 5.9	17.6 ± 5.9	*p* < 0.001
STAI (score)	33.2 ± 9.3	32.5 ± 9.0	*p* < 0.05
sAA (U/ml)	61.8 ± 27.0	56.2 ± 28.5	*p* < 0.05

### 4.4 Correlation among scale scores

Correlations between the subscales ‘A state that makes you laugh,’ ‘Actively making others laugh,’ and ‘Forced laughter’ of the Laughter Attitude Scale and the pre-CLP Optimism and Pessimism scores were confirmed (Table 5). A strong negative correlation of – 0.72 (*p* < 0.001) was found between optimism and pessimism scores, whereas a moderate correlation of – 0.56 (*p* < 0.001) was found between the optimism score and STAI, and 0.57 (*p* < 0.001) between the pessimism score and STAI. There was a strong correlation between ‘A state that makes you laugh’ and each of ‘Actively making others laugh’ (0.83, *p* < 0.001) and ‘Forced laughter’ (0.47, *p* < 0.001). There was no significant correlation between ‘Forced laughter’ and ‘Actively making others laugh.’ There was a weak correlation between ‘A state that makes you laugh’ and STAI (– 0.29, *p* < 0.001), and a weak positive correlation between the optimism score and each of ‘A state that makes you laugh’ (0.35, *p* < 0.001) and ‘Actively making others laugh’ 0.32 (*p* < 0.001). sAA was not significantly correlated with any of the scores, including the STAI.

**Table 5.** Correlation among each scale score (n = 101).

	A state that makes you laugh easily		Actively Making someone laugh		Forced laughter		Optimism		Pessimism		STAI	sAA
A state that makes you laugh easily	–											
Actively making someone laugh	0.83	**	–									
Forced laughter	0.46	**	0.11		–							
Optimism	0.36	**	0.33	**	−0.04		–					
Pessimism	−0.17		−0.17		0.09		−0.72	**	–			
STAI	−0.3	**	−0.3	**	0.01		−0.57	**	0.56	**	–	
sAA	−0.04		−0.01		−0.1		0.04		0.13		0.12	–

Statistical Analysis: Spearman r correlation **p < 0.01.

### 4.5 Factors predictive of post optimism score

The regression model with the optimism score as the dependent variable (n = 103) identified pre pessimism score (β =  – 0.28, *p* < 0.001), ‘A state that makes you laugh’ (β = 0.46; *p* < 0.001), ‘Forced laughter’ (β =  – 0.3, *p* < 0.001), and pre STAI (β =  – 0.34, *p* < 0.001) as factors significantly influencing optimism score (see Table 6). The model explained 50% (adjusted R2) of the variance in optimism.

**Table 6.** The predictive factors of post optimism score (n = 101).

Predictor variables	Standardized coefficients β	t	*p*
age	−0.015	−0.201	0.838
gender	0.005	0.08	0.96
Frequency of laughing	0.009	0.13	0.9
Pessimism (pre)	−0.37	−4.32	<0.001
A state that makes you laugh	0.65	3.51	0.001
Actively making others laughter	−0.24	−1.53	0.128
Forced laughter	−0.32	−2.92	0.004
STAI(pre)	−0.26	−2.88	0.005

Full model: R2 = 0.55; *p* < 0.001, Significant result (*p* < 0.001)

^a^ Dependent variable: optimism.

## 5. Discussion

The present study examined whether watching a comedy stage performance had increased optimism, which is known to be beneficial for physical and mental health and for reducing pessimism, anxiety, and sAA. The major findings were that optimism was significantly higher, and that pessimism, STAI and sAA were significantly lower, when compared before and after the CLP. The CLP featured four comedians that have been popular in Japan for over five years. Although a large percentage of the participants were in their 30s to 50s, the comedians are all talented performers with consistently high media exposure and popularity among a wide range of people, and the entire audience was constantly drawn into laughter. About 90% of the participants enjoyed the performance very much and laughed a lot. The survey responses revealed that optimism increased and pessimism, anxiety and stress level decreased as a result of enjoying the two-hour-plus CLP.

It is well known that the secretion of sAA increases to reflect the sympathetic nervous function that is enhanced by stress (Skosnik et al., 2000; Takai et al., 2004). sAA has a faster response to stress than salivary cortisol, and is recently attracting attention as an objective stress assessment method (Keremi et al., 2017; Yamaguchi et al., 2005). Since this response occurs in a short time through directly sympathetic nerve, it occurs in a short period of time, and many reports that take advantage of this characteristic (Noto et al., 2005; Walther et al., 2022; Wyss et al., 2016). On the other hand, there are also several reports regarding the relationship between chronic stress and sAA (Booij et al., 2015; Cozma S et al., 2017). Furthermore, Takai et al. found that sAA increased with unpleasant stimuli, but decreased with pleasant stimuli, suggesting that sAA is an indicator of sedation or relaxation (Takai et al., 2004). In this study showed that over half the people (63.7%) had a decrease in sAA after the CLP. There are probably that attending a CLP may the suppression of sympathetic nerve excitation resulting from chronic psychological stress. Whereas it is clear that there is a significant increase in the sympathetic nervous system with laughter, a decrease in the sympathetic nervous system with recovery, and a significant increase in the parasympathetic nervous system, guiding a relaxing effect (Yamada & Ayabe, 2022). sAA release is induced by activation of the sympathetic nervous system, and since the response time is short, ranging from 1 to several minutes, it can be inferred that the activation of the parasympathetic nervous system causes a decrease in sAA after the CLP. In addition, although some previous studies have shown that the higher the sAA, the higher the anxiety, there was no significant association between STAI and sAA in this study. Although anxiety is related to stress regulation in the amygdala (Shioiri, 2012), it is not possible to explain the reduced stress levels induced by CLP solely by a reduction in sAA in the sympathetic nervous adrenal medullary system (SAM). It is also necessary to examine the hypothalamic pituitary adrenal (HPA) system of cortisol by a slightly delayed response, including cortisol, and it is possible that the relationship with STAI and sAA was not clearly demonstrated.

As far as optimism goes, it has been reported that optimism is the most influential factor in mental health among people of Asian ethnicity (Chang, 1996), and the higher optimism and the lower pessimism in this empirical study therefore indicates that CLP can contribute to mental health. Optimism and pessimism can be conceptualized in various ways, being regarded as one-dimensional in some studies (Rauch et al., 2007) and two-dimensional in others (Herzberg et al., 2006). The results of the present analysis indicate that optimism and pessimism changed in a one-dimensional manner after the CLP.

To clarify the factors that influence optimism after a CLP, we conducted multiple regression analysis using pessimism gain and STAI, subscales of the Laughter Attitude Scale, age, gender, and frequency of laughter as independent variables. Significant standard partial regression coefficients were found for ‘A state that makes you laugh’ (0.65), forced laughter (– 0.32), STAI (– 0.26), and pessimism (– 0.37), in that order. In terms of attitude toward laughter, those who tended to be made to laugh by others and those who tended not to smile affectionately were more likely to be optimistic. Being made to laugh by others and actively making others laugh were strongly correlated, as described above, and high attitude toward laughter was a predictor of optimism. At the same time, low anxiety, low pessimism, and no affectionate laughter were also predictors of optimism. It has previously been reported that a low level of optimism is a predictor of high anxiety levels (Faye-Schjoll & Schou-Bredal, 2019); however, the opposite was found in the present study. In addition, forced smile consists of emotion-control forced laughter, which resolves or conceals unpleasant or sad feelings; atmosphere-control forced laughter, which is used to create an atmosphere to soothe others; and behavior-control forced laughter, which is used to try to control the behavior of others (Oshimi, 2000) and is strongly associated with as a cover-up of emotions to avoid negative social evaluation (Oshimi, 2002). Forced laughter can be considered an extremely social psychological behavior and works in opposition to the natural enjoyment of humor and the enhancement of positive thinking. Although not included in the factors examined in this study, it is clear that people with high optimism are highly social and able to build close relationships with others (Carver et al., 1994; Helweg-Larsen et al., 2005), and it can be assumed that people who laugh often and are optimistic by nature have strong social connections. On the other hand, because the frequency of meeting people increases significantly by laughter intervention, laughter is suggested to improve not only well-being but also social functioning (Funakubo et al., 2022). The most expected benefits of laughter are for groups with low social interaction, isolation, and pessimism. A single intervention may not be sufficient, and repeated efforts and long-term efforts involving social interaction will be important.

There are some limitations of the present study. Bias exists because only the groups who volunteered to participate in the demonstration experiment were included, and these are not representative of the population. In addition, the intervention was a one-time event, the long-term effects of the intervention, and whether or not they participated in the group, are unknown. In addition, the timing of the measurements may have affected the stress markers.Furthermore, detailed background information of the participants, such as pre-existing medical conditions, family environment, and economic situation, are unknown. Although it is possible that some of these could have affected the results, there are limitations in considering these factors. Despite these limitations, we consider that repeated efforts of municipalities to increase the amount of laughter in the entire community and foster a society in which people laugh will lead to improvements in physical health, mental health, and the revitalization of the community. In order to generate evidence supporting the integration of CLP into clinical practice and health policy, it is hoped that randomized intervention studies will be conducted in the future to clarify its effectiveness.

## 6. Conclusions

CLP is an inexpensive and accessible form of entertainment that everyone can enjoy, and its contribution to health benefits is significant. This study demonstrated that two hours of CLP increased optimism and decreased pessimism, anxiety and stress levels among attendees. Factors contributing to optimism were strongly influenced by the degree to which people made them laugh, as well as pessimism, anxiety, and low levels of forced laughter. These results suggest the need for further research with long-term intervention studies and controlled study design to better understand the effects of laughter.

## Institutional review board statement

The study was conducted in accordance with the Declaration of Helsinki and was approved by an Institutional Review Board/Ethics committee. See details under Methods.

## Data Availability

To protect participant privacy and prevent the re-identification of individuals from some quantitative data, individual participant data will not be shared. The questionnaire and raw data are available upon request.
